# Post-traumatic headache: the use of the sport concussion assessment tool (SCAT-3) as a predictor of post-concussion recovery

**DOI:** 10.1186/s10194-017-0767-5

**Published:** 2017-05-30

**Authors:** Olivia Begasse de Dhaem, William B. Barr, Laura J. Balcer, Steven L. Galetta, Mia T. Minen

**Affiliations:** 1 Department of Internal Medicine, New York University Langone Medical Center, New York, NY USA; 2 Department of Neurology, Columbia University – New York Presbyterian Hospital, New York, NY USA; 3 Department of Neurology, New York University Langone Medical Center, 240 East 38th Street, New York, NY USA

**Keywords:** Evaluation, Post-traumatic headache, SCAT-3, Concussion screening, Symptom severity, Head injury

## Abstract

**Background:**

Given that post-traumatic headache is one of the most prevalent and long-lasting post-concussion sequelae, causes significant morbidity, and might be associated with slower neurocognitive recovery, we sought to evaluate the use of concussion screening scores in a concussion clinic population to assess for post-traumatic headache.

**Methods:**

This is a retrospective cross-sectional study of 254 concussion patients from the New York University (NYU) Concussion Registry. Data on the headache characteristics, concussion mechanism, concussion screening scores were collected and analyzed.

**Results:**

72% of the patients had post-traumatic headache. About half (56.3%) were women. The mean age was 35 (SD 16.2). 90 (35%) patients suffered from sport-related concussions (SRC). Daily post-traumatic headache patients had higher Sport Concussion Assessment Tool (SCAT)-3 symptom severity scores than the non-daily post-traumatic headache and the headache-free patients (50.2 [SD 28.2] vs. 33.1 [SD 27.5] vs. 21.6 SD23], *p* < 0.001). Patients with SRC had lower headache intensity (4.47 [SD 2.5] vs. 6.24 [SD 2.28], *p* < 0.001) and SCAT symptom severity scores (33.9 [SD 27.4] vs. 51.4 [SD 27.7], *p* < 0.001) than the other patients, but there were no differences in post-traumatic headache prevalence, frequency, and Standardized Assessment of Concussion (SAC) scores.

**Conclusion:**

The presence and frequency of post-traumatic headache are associated with the SCAT-3 symptom severity score, which is the most important predictor for post-concussion recovery. The SCAT-3 symptom severity score might be a useful tool to help characterize patients’ post-traumatic headache.

## Background

Traumatic brain injury (TBI) is a major cause of disability. The estimated 5.3 million Americans living with TBI-related disability face numerous challenges in their efforts to return to a full and productive life [1]. Post-traumatic headache is one of the most prevalent post-concussion sequelae (up to 89% of patients suffer from it), is one of the longest lasting post-concussion symptoms, causes significant morbidity, and might be associated with slower neurocognitive recovery [2–5].

Despite the plethora of complaints associated with traumatic brain injury and headache being the most prevalent complaint, there are no standard guidelines or recommendations for post-traumatic headache evaluation and management [6, 7]. Although patients who suffered from severe TBI might become aware of their post-traumatic headache more than seven days after the accident once their mental status improve and the post-traumatic headache severity of patients who suffered from mild TBI improves over time, the optimal timing for post-traumatic headache assessment has not been evaluated yet [8, 9]. However, there are validated tools to evaluate post-concussion signs and symptoms in athletes, which are now being implemented for use in non-athlete populations. The Sport Concussion Assessment Tool (SCAT-3) was initially developed at the Second International Conference on Concussion in Sport in Prague in 2004 by combining existing tools such as the Glasgow coma scale (GCS), modified Maddocks Score, modified Post-Concussion Symptom Scale (PCSS), mechanism of injury and background information, Standardized Assessment of Concussion (SAC), and examinations of the neck, balance, and coordination [10, 11]. The GCS is an assessment of patient’s levels of consciousness [12]. The Maddocks Score is an assessment of athletes’ orientation after traumatic brain injury [13]. The PCSS is a self-reported measure of 21 symptoms (representing four categories: somatic, cognitive, emotional, and sleep) weighted on a 7-point Likert severity scale [14]. The SAC is a rapid evaluation of patients’ orientation, concentration, and immediate and delayed memory [15]. Thus, the SCAT-3 arose from expert consensus and combines both the post-concussion symptom checklist and a quick set of examination tasks to assess some of the signs often comorbid with posttraumatic headaches such as cognitive or balance deficits. A longitudinal study of high school and college athletes indicates that the acute SCAT-3 symptom severity score is the most sensitive and significant predictor of recovery from SRC and hence of functional impairment and service utilization [16]. The King-Devick test (K-D) requires saccadic eye movements, language and other cognitive processes to perform [17]. However, a limitation of the SCAT-3 and the K-D is that they were designed for athletes, while the primary cause of TBIs in patients seen in the Emergency Department are falls, being struck by/against, motor vehicle accidents, and assaults [18]. Athletes tend to underreport their concussion and thus may not be representative of the general population [19]. Thus, additional study is required to determine the usefulness of the standardized concussion evaluation tools in non-athlete patients to screen for those who might benefit most from aggressive post-traumatic headache management to help with post-concussion syndrome recovery and prevent pain sensitization.

Since the SCAT-3 symptom severity score is the most significant predictor of recovery from SRC in athletes and since headache is the most common post-TBI symptom and is included in the SCAT-3 symptom severity score, we hypothesize that the SCAT-3 symptom severity score could be used to evaluate post-traumatic headache in athletes and non-athletes. We conducted two-tailed Student’s t-test of the SCAT-3 symptom severity scores between headache-free patients and post-traumatic headache patients with a criterion for significance of *p* < 0.05). Little is known about the non-athlete post-TBI recovery prognostic indicators [20]. Assessing a potential relationship between SCAT-3 scores and post-traumatic headache pain intensity and frequency (defined as daily versus not daily) might help plan post-traumatic headache management and prognostic recovery based on the SCAT-3 symptom severity scores. We sought to 1) assess the relationship between headache intensity, frequency, and concussion screening scores (SCAT-3 and King-Devick [K-D]), 2) compare these scores and post-traumatic headache of patients with and without SRC, and 3) determine whether the reported SCAT-3 concussion symptoms have a relationship to the formal assessments in our heterogeneous sample of patients.

## Methods

### Participants

The New York University (NYU) School of Medicine Institutional Review Board approved the study (IRB number S13–01229) on 8/16/2013. This is a retrospective cross-sectional study of the 254 concussion patients whose data was entered into the NYU Concussion Registry. Concussion was defined as a new neurologic symptom or sign that follows an impulsive blow to the head or body that was otherwise unexplained. Data were abstracted from office visits between 7/29/2011 to 8/17/2015 (when this study commenced); a sample size was not calculated for this study. The dates of the patients’ head injuries for which they were referred to the NYU Concussion Center range from 9/11/2000 to 7/30/2015. They were referred to the NYU Concussion Center between the day of concussion to 4774 days later (mean 131 days, SD 378, median 25 days, IRQ 81.5). Some results are missing from the registry due to differences in the timing of the introduction of some of the tests for concussion patients in our Concussion Center, because patients were unable to perform certain tests such as the balance exam, refused to do them, or did not have time to complete the full evaluation. Prior studies have been published using this data registry [21–23].

### Method

The patients referred to the NYU Concussion Center were asked to complete a baseline questionnaire that included questions about demographics, prior concussions, prior injuries, and participation in sports. They were assessed by trained physicians using a modified PCSS reported as the SCAT-3 symptom and symptom severity score, the Standardized Assessment of Concussion (SAC), a modified Balance Error Scoring System (BESS), and the K-D. The SCAT-3 symptom evaluation is a modified version of the PCSS. It is recorded as the SCAT-3 symptom score, which is the number of self-reported symptoms out of a 22-item list, and as the total SCAT-3 symptom severity score which is weighted based on a 7-point Likert scale of severity of these symptoms [24]. The Likert scale starts at 0 and the maximum score is 132 based on 6 being the maximum score for any one symptom and there are 22 possible symptoms. SAC is a mental status test assessing the patients’ orientation, immediate memory, delayed memory, and concentration with a maximal possible total score of 30 [24]. The BESS is a test of balance assessing six stances. One point is given for every error, so high scores suggest poor performance [24]. The K-D test is a rapid number naming test to assess saccadic eye movements that are required for reading. Patients are asked to read single-digit numbers from left to right on different cards as fast as possible making the least errors possible. Since only 11 of our patients made an error, we used their time as the main measure of their K-D scores [24]. Patients were also asked about the presence of headache prior and after their concussions, the intensity of their headaches (recorded using the 11-item Numeric Rating Scale for pain), and their frequency (daily versus not daily) [25]. The study data were collected and managed using REDCap Software Version 6.10.14 electronic data capture tools hosted at NYU School of Medicine [26].

### Statistical analysis

Our predictors were post-traumatic headache presence, intensity, and frequency. Our primary outcome was the SCAT-3 symptom severity score. Secondary outcomes included SCAT-3 symptom score, and other concussion scores such as the SAC, BESS, and K-D. Student’s t-test, ANOVA, Chi-square test, linear regressions, Shapiro-Wilk test and Mann U Whitney test were performed. Statistical analyses were conducted with Excel V15.0.4833.1001 and IBM SPSS Statistics V22.0.0.

## Results

### Baseline characteristics

As shown in Table 1, among the 254 concussion patients enrolled in the study, 56% were women. The mean age was 35 years of age. About one eighth (15%) suffered from headaches prior to their concussions and about three quarter (72%) developed new headaches after their concussions. About two third (66%) denied a prior history of concussion. About one third (35%) of patients suffered from sport-related concussions; the rest were injured from falls, motor vehicle accidents, and another trauma. There was a lot of missing data on the concussion assessment test results. The specialists generally offered every patient the same tests. However, as stated above, some results were missing because subjects were unable to perform certain tests such as the balance exam, refused to do them, or time precluded full evaluation. Out of 254 patients, 117 results were missing for the K-D test, 118 for the SAC, 145 for the BESS (mostly reported as “unable to tolerate” or “unable to perform”), and 125 for the SCAT-3 symptom scores. Of note, the missing data involve the same overlapping patients except for the few extra missing data in certain groups. Also, the severity of the reported SCAT-3 symptoms corresponded with the BESS and SAC scores in our analysis. As per Table 2, the characteristics of the group with missing test result data did not differ from our sample.

**Table 1:** Sample characteristics

Demographics/history	Mean (SD) or number of subjects (%)
Total Number of Subjects	254
Female	143 (56.3%)
Age (years)	34.6 (SD 16.2, median 31)
Prior headache history	38 (15.0%)
Post-traumatic headache	183 (72.1%)
Prior concussions	
0	66.1%
1	10.7%
2	12.1%
3+	11.2%
Mechanism of injury	
Falls	32.7%
Motor vehicle accident	21.3%
Assault	5.12%
Other trauma	5.52%
Sports-related	35.4%
Time between concussion and assessment (days)	mean 131 (SD 379, median 25)
Loss of consciousness	69 (27.2%)
Amnesia	42 (16.5%)

**Table 2:** Characteristics of the group for which missing data test results

Demographics/history	Mean (SD) or number of subjects (%)
Total Number of Subjects	117
Female	48 (41%)
Age (years)	37.8 (16)
Post-traumatic headache	93 (79.5%)
Mechanism of injury	
Falls	30%
Motor vehicle accident	18%
Assault	4.27%
Sports-related	27.4%
Time between concussion and assessment (days)	135 (378)

### Headache history

A prior history of headaches is not associated with any of the concussion scores assessed such as the SCAT-3 symptom severity score (45.8 [SD 28.5] vs. 43.2 [SD 30.1], *p* = 0.69), SCAT-3 symptom score (14.7 [SD 6.1] vs. 14.4 [SD 7], *p* = 0.83), SAC (26.8 [SD 2.7] vs. 26.5 [SD 2.1], *p* = 0.58), BESS (2.88 [SD 4] vs. 7.14 [SD 9.6], *p* = 0.084), and K-D (64.7 [SD 23.3] vs. 54.9 [SD 22.2], *p* = 0.080).

### Concussion history

Table 3 shows that in our sample, a concussion history is not associated with post-traumatic headache prevalence (χ2 *p* = 0.092), headache intensity (Student’s t-test *p* = 0.60), nor SCAT-3 symptom severity and symptom scores (*p* = 0.53).

**Table 3:** Concussion history and headache prevalence, headache severity, and SCAT-3 symptom scores

	No prior concussion	Prior concussion	statistic
Post-traumatic headache prevalence	148 (84.6%)	72 (92.4%)	Chi-square *p*-value 0.092
Post-traumatic headache severity	5.6 (SD 2.5), *N* = 80	5.4 (SD 2.6), *N* = 39	2 tailed t-test *p*-value 0.60
SCAT-3 symptom severity score	42.0 (SD 28.6), *N* = 67	45.3 (SD 28.0), *N* = 51	2 tailed t-test *p*-value 0.53
SCAT-3 symptom score	14.2 (SD 6.11), *N* = 67	14.9 (SD 6.46), *N* = 51	2 tailed t-test *p*-value 0.53

Of note, performing an ANOVA for post-concussion headache severity based on number of prior concussions did not show any difference, but the sample size for each category was low.

### Post-traumatic headache and SCAT-3 symptom severity and SCAT-3 symptom scores

**Fig. 1:**
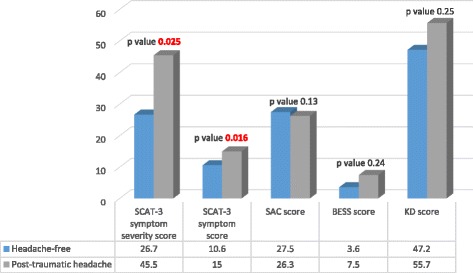
SCAT-3, SAC, BESS, and KD Scores in Headache-Free Patients vs. Post-Traumatic Headache Patients

**Fig. 2:**
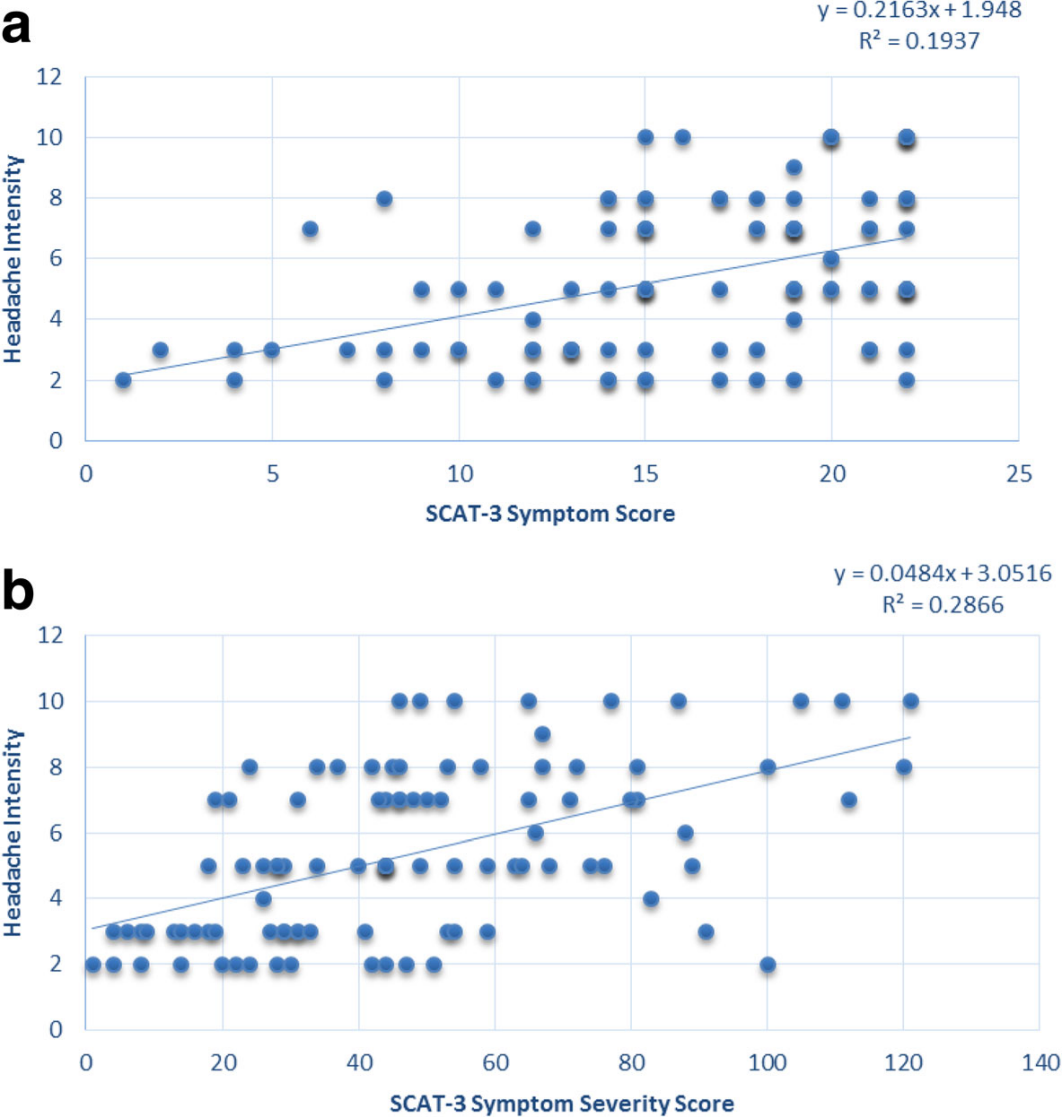
Linear regressions of SCAT-3 symptom and symptom severity scores and post-traumatic headache intensity. **a** Linear regression of SCAT-3 symptom score and post-traumatic headache intensity. **b** Linear regression of SCAT-3 symptom severity score and post-traumatic headache intensity

**Fig. 3:**
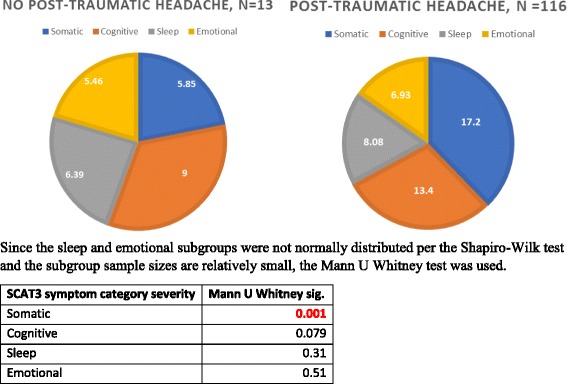
Partial SCAT-3 symptom scores by symptom categories in headache-free vs. post-traumatic headache patients

Figure 1 shows that the SCAT-3 symptom score and the SCAT-3 symptom severity score are both significantly higher in post-traumatic headache patients than in headache-free patients (15.0 [SD 5.89] vs. 10.6 [SD 6.73], *p* = 0.016; 45.5 [SD 28.7] vs. 26.7 [SD 21.1], *p* = 0.025). Table 4 shows that daily post-traumatic headache patients had higher SCAT-3 symptom score and higher SCAT-3 symptom severity scores than the non-daily post-traumatic headache and the headache-free patients (16.2 [SD 5.2] vs. 11.7 [SD 7.1] vs. 8.33 [SD 6.6] *p* < 0.001; 50.2 vs. 33.1 vs. 21.6, *p* < 0.001). Figure 2 shows no significant associations between post-traumatic headache intensity and SCAT-3 symptom and SCAT-3 symptom severity scores (correlation coefficient of 0.22 and R2 of 0.19, and of 0.048 and 0.29 respectively)). Table 5 shows that post-traumatic headache patients have higher SCAT-3 symptom and symptom severity scores than headache-free patients even when not including the headache symptom score when calculating these two SCAT-3 symptom scores. Figure 3 shows that post-traumatic headache patients have significantly more severe somatic symptoms than headache-free patients [27]. However, no difference is observed between their cognitive, emotional, and sleep severity scores.

**Table 4:** SCAT-3, SAC, BESS, and K-D scores in headache-free patients, non-daily post-traumatic headache patients, and daily post-traumatic headache patients

Mean (SD)	No headache	Non-daily post traumatic headache	Daily post-traumatic headache	ANOVA p
SCAT3 symptom severity score	21.6 (23), *N* = 9	33.1 (27.5), *N* = 33	50.2 (28.2), *N* = 84	**<0.001**
SCAT3 symptom score	8.33 (6.6), *N* = 9	11.7 (7.1), *N* = 33	16.2 (5.2), *N* = 84	**<0.001**
SAC score	28 (3.4), *N* = 10	26.7 (2.1), *N* = 31	26.5 (2.8), *N* = 92	0.18
BESS score	2.71 (4), *N* = 7	6.67 (7.1), *N* = 27	6.82 (9.8), *N* = 73	0.53
KD score	45.5 (17.9), *N* = 7	53.8 (23.8), *N* = 32	57.8 (24.8), *N* = 96	0.29

The statistically significant results were highlighted in bold

**Table 5:** SCAT-3 symptom and symptom severity scores without taking headache into account in the calculation of the scores in headache-free patients vs. post-traumatic headache patients

	No post-traumatic headache Mean (SD)	Post-traumatic headache Mean (SD)	2-tailed t-test
SCAT-3 symptom score without counting headache	10.6 (6.73), *N* = 13	14.0 (6.01), *N* = 116	0.063
SCAT-3 symptom severity score without counting headache	26.2 (20.9), *N* = 13	42.8 (28.1), *N* = 116	**0.041**

The statistically significant results were highlighted in bold

### Post-traumatic headache and SAC, BESS, and K-D scores

Figure 1 and Table 4 indicate that there are no differences in SAC, BESS, and K-D scores between headache-free patients and post-traumatic headache patients regardless of post-traumatic headache frequency.

### Post-traumatic headache and concussion scores in patients with and without sport-related concussion (SRC)

**Fig. 4:**
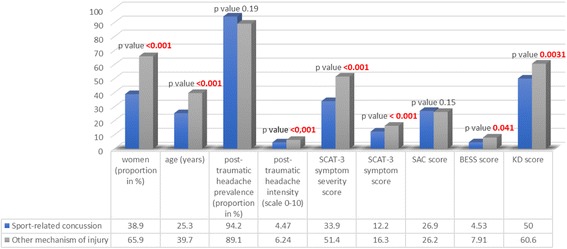
Post-traumatic headache and concussion scores in patients with and without sport-related concussion

Figure 4 indicates that SRC patients are significantly younger than the non-sport related concussion patients. In addition, the SRC patients are predominantly men (61.1%) whereas the other group is predominantly women (65.9%). SRC patients have significantly better K-D scores (50.0 [SD 15.1] vs. 60.6 [SD 25.8], *p* = 0.003), better BESS scores (4.53 [SD 6.69] vs. 7.91 [SD 19.3], *p* = 0.041), milder post-traumatic headache intensity (4.47 [SD 2.5] vs. 6.24 [SD 2.28], *p* < 0.001), and lower SCAT symptom and symptom severity scores (33.9 [SD 27.4] vs. 51.4 [SD 27.7], *p* < 0.001)than the other patients. There is no association for post-traumatic headache prevalence, frequency, and SAC scores.

### SCAT-3 reported symptoms vs. formal assessments in our heterogeneous sample

Linear regression analyses indicate a positive association between severity of the self-reported balance difficulty and modified BESS score (R2 = 0.95), between the self-reported difficulty concentrating severity score and the SAC concentration score (R2 = 0.36), between the self-reported concentration difficulty severity and the SAC immediate memory score and delayed recall (R2 = 0.99 and 0.66). Although an increased reported severity seems to be associated with a worse SAC concentration score, the difference in SAC concentration scores (3.3–3.8 range) was very small compared to the difference in reported severity (1–4 range). Given all patients did very well for the SAC scores, especially for the SAC immediate memory score (13.7–14.6 / 15), it is unclear whether this association would be of clinical importance.

## Discussion

In this heterogeneous population who presented to an outpatient multidisciplinary concussion center (heterogeneous in terms of age, gender, and mechanisms of injury), we have several findings regarding the use of SCAT-3 in post-traumatic headache patients: 1. The presence and frequency of post-traumatic headache are associated with the SCAT-3 symptom severity score, especially with the somatic SCAT-3 symptoms. 2. The SCAT-3 symptom scores might be a useful tool for neurologists and headache specialists as they incorporate balance, concentration, and memory. The self-reported symptoms of balance, concentration, and memory correlate with the formal SCAT-3 assessments and subscores in our sample.

As indicated above, our sample population is heterogeneous in terms of age, gender, and mechanism of injury. A little more than a third suffered from sport-related concussions. Although the data on the gender-specific prevalence of TBI is somewhat contradictory, it is interesting that TBI was more prevalent in women than men in our sample of non-athlete civilian patients [28, 29]. In our sample, men had a higher rate of sport-related concussion while women tended to have other mechanisms of injury. In our sample, a prior history of neither headache nor concussions is associated with the prevalence and intensity of posttraumatic headaches, but our sample size for the patients with a prior history of concussion is not large enough for adequate power. This is in contradiction with reports that found a history of mild TBI is associated with a higher prevalence of migraine-like headaches [30]. and a history of prior TBIs has also been found to correlate with post-traumatic headache prevalence [23].

Both post-traumatic headache prevalence and frequency are associated with higher SCAT-3 symptom severity and SCAT-3 symptom scores. This is important because the SCAT-3 symptom severity score is the most sensitive and significant predictor of recovery from SRC [19, 31]. Post-traumatic headache patients have higher SCAT-3 symptom scores than headache-free patients regardless of whether the “headache” symptom is counted when calculating the SCAT-3 symptom scores. Interestingly, when comparing SCAT-3 symptoms for each of the four symptom categories individually, post-traumatic headache patients have significantly higher somatic SCAT-3 symptoms, but a similar occurrence and severity of sleep, emotional, and cognitive symptoms compared to the headache-free patients. Of note, the somatic symptoms of the SCAT-3 assessment (e.g. blurred vision, photophobia, phonophobia, neck pain, nausea, vomiting) are often associated with headaches. This might explain the association between post-traumatic headache and the somatic SCAT-3 symptoms. Another explanation would be that post-traumatic headache patients have higher somatization than headache-free patients. Prior literature suggests that somatization is associated with higher SCAT-3 symptom severity scores and longer recovery [32].

When comparing the subjective and objective assessments, in our heterogeneous sample of patients with diverse mechanisms of injury who presented to the concussion center, the SCAT-3 self-reported symptoms of balance, concentration, and memory correlate with the modified BESS and SAC scores, which supports the use of these scores among our heterogeneous population of concussion center patients who are not necessarily athletes.

The SRC patients in our sample have less severe post-traumatic headache and lower SCAT-3 symptom scores than the non-sport-related concussion patients, for the same SAC scores –which is a marker of effort- [24]. The difference in headache severity and SCAT-3 symptom scores might be at least partially confounded by the significant difference in age, gender, BESS, and/or K-D scores between the two groups. Indeed, a prior study on concussion assessments in an outpatient heterogeneous population like ours showed that older age and female gender are associated with worse SCAT-3 symptom scores [23]. Interestingly, our group with the worse SCAT-3 symptom scores (the non-sport related concussion group) is older with a higher proportion of women than the SRC group. Further studies with a larger sample would be helpful to adjust for age, gender, BESS, and K-D scores.

Although we need further studies to compare post-traumatic headache and the SCAT-3 symptoms between SRC patients and patients with concussions unrelated to sports, the SCAT-3 seems to be a good tool to evaluate post-traumatic headache in the general population of post-concussion patients.

## Limitations

Our study had several limitations. One limitation was that we had a high number of patients with headache, thus making it hard to compare post-traumatic headache patients with headache-free patients. A second limitation is the retrospective nature of the study and its missing data. The missing data is likely multifactorial including the introduction of the different concussion tests at different time and some patients feeling too symptomatic to perform full testing. The missing data was handled with pairwise deletion. A third limitation was the wide range of time to assessment which could contribute to recall bias in that patients with mild headaches closer to the time of concussion onset may have under-reported a prior history of headaches. Very few patients were assessed within the first seven days and it is known that symptoms gradually resolve by post-concussion day 7 on average in SRCs in athletes [33]. A fourth limitation was that, if a concussion was severe, patients with a prior history of headaches may have dismissed their prior history of headaches and denied having a prior history of headaches. Having severe symptoms acutely is a risk factor for severe symptoms later on, and vice versa, having severe symptoms later on suggests having had severe symptoms early on. A fifth limitation was that our study suffers from selection bias. Our study participants willingly sought care at our tertiary referral center, so their TBI symptoms might be worse than for those who did not seek treatment. Some also might have sought care somewhere else first, which might have confounded our results. Interestingly, a large portion of our patients were evaluated at our center more than three months after their concussions and the patients who presented to our center within three months of their concussions reported less severe headaches than the patients who presented to our center more than three months after their concussions, which might illustrate a selection for patients with more severe symptoms in our sample. Patients who present earlier tend to present because they are worried they hit their heads. Patients who present on the later side are concerned about their persistent symptoms. A sixth limitation is that we do not have pre-injury scores. Concussion scores are most helpful when compared pre- and post-injury in the same patients. A seventh limitation is due to the fact that registry data was used for a report of headache following concussion (what we term “post-traumatic headache”)-not physician assessments using the International Classification of Headache Disorders (ICHD) 3 beta criteria. Therefore, we do not know if the headaches occurred within seven days of the injury or being off of medication which could have masked headaches from the injury [34]. Also, we are limited by the data from the assessments and the physician notes, and thus do not have the mean number of headache days.

## Future studies

It would be helpful to compare post-traumatic headache and SCAT-3 symptom scores between athletes and non-athletes adjusting for age, gender, BESS, and K-D scores. Since the SCAT-3 symptom severity score is an important prognostic indicator of post-concussion recovery and is associated with post-traumatic headache, it would be interesting to evaluate the impact of early post-traumatic headache management on post-concussion recovery and SCAT-3 symptom severity score.

## Conclusions

Most of our post-concussion patients suffered from post-traumatic headache. Both post-traumatic headache prevalence and frequency are associated with higher SCAT-3 symptom severity and symptom scores. The association between post-traumatic headache and SCAT-3 symptom severity score stems from higher SCAT-3 somatic symptom scores in post-traumatic headache patients, which might reflect either a higher degree of somatization in post-traumatic headache patients or an overlap between the SCAT-3 somatic symptoms and symptoms often associated with headaches. SCAT-3 somatic symptoms are important in concussion management and recovery prognosis. Since the SCAT-3 symptom severity score seems to be the single most important predictor for post-concussion recovery and since SCAT-3 symptom severity score is associated with post-traumatic headache, the SCAT-3 symptom severity score seems to be a promising standardized tool to identify the post-concussion patients who might benefit from additional follow-up and management for post-traumatic headache and associated symptoms. It was shown that standardized measurement of post-concussive symptoms including headache may enhance clinical management of concussion patients. Although the SCAT-3 was designed for athletes, our study suggests that it might be helpful in the general population of concussion patients to assess for post-traumatic headache and associated symptoms.
